# Supplementary material for: Validation of Recycled Nanofiltration and Anion-Exchange Membranes for the Treatment of Urban Wastewater for Crop Irrigation
Source: Membranes (Basel). 2022 Jul 29;12(8):746. doi: 10.3390/membranes12080746 (PMC9412259; doi:10.3390/membranes12080746)
Supplement: Supplementary file 1 [file membranes-12-00746-s001.zip › membranes-1835077-supplementary.pdf]

Supplementary Material

for

Validation of recycled nanofiltration and anion-exchange membranes for the treatment of high salinity urban wastewater for crop irrigation

Anamary Pompa-Pernía[1,2,*], Serena Molina[1], Amaia Lejarazu-Larrañaga[1], Junkal Landaburu-Aguirre[1], Eloy García- Calvo[1,2]

[1] IMDEA Water Institute, Avenida Punto Com, 2, Alcalá de Henares, 28805 Madrid, Spain

[2] Chemical Engineering Department, University of Alcalá, Ctra. Madrid-Barcelona Km 33.600, Alcalá de Henares, 28871 Madrid, Spain

* Correspondence to:

anamary.pompa@imdea.org

S1. Characterization of recycled nanofiltration membranes

Some preliminary tests were carried out by the research team once the membrane was transformed to ensure that nanofiltration properties were achieved. Considering to replicate and compare with the previous studies conducted by our research group, it was used the same synthetic brackish water (Table S1) and tested under the same conditions. Previously, the end-of-life hydraulic permeance was measured using Milli-Q water.

Table S1. Average water quality parameters of the synthetic brackish water.

Conductivity (µm/cm)	6,225±487	Cl^- (ppm)	1,273±56	Na^+ (ppm)	804±42
pH	6.4±0.3	SO_4^{2-} (ppm)	1,651±102	Mg^{2+} (ppm)	409±26

The end-of-life RO membrane showed permeance of 5.95 L m^{-2} h^{-1} bar^{-1}. In contrast, after the exposure dose of 8 000 ppm h oxidizing agent, the rNF membrane reached a permeance of 13.95 L m^{-2} h^{-1} bar^{-1}. Regarding the rejection percentage of salts, the studied membrane showed 63.34 % of total salt rejection. Though individual ion rejections resulted in 50.64 % for Cl^-, 56.61 % for Na^+, 89.92 % for SO_4^{2-}, and 83.79 % for Mg^{2+}. These results verified that NF permeance properties were reached.

Similar results were reached in the previous recycled NF membranes study carried out by our research group. The achieved performance was similar to the performance of NF commercial membranes (NF270 and NF90 from Dow), both measured under the same conditions. An overview of these previous results can be found in [1].

Attenuate total reflectance-Fourier transform infrared

Membranes exposed to NaOCl were characterized by ATR-FTIR spectroscopy using a Perkin-Elmer RX1 spectrometer equipped with an internal reflection element of a diamond at an incident angle of 45°. An adequate pressure was applied to the membrane placed on the crystal surface. The spectra were recorded at a resolution of 2.0 cm^{-1} in the frequency region of 4 000 – 650 cm^{-1}, with an average of 4 scans per sample. Previously the samples were dried at 100 °C to remove moisture for two days.

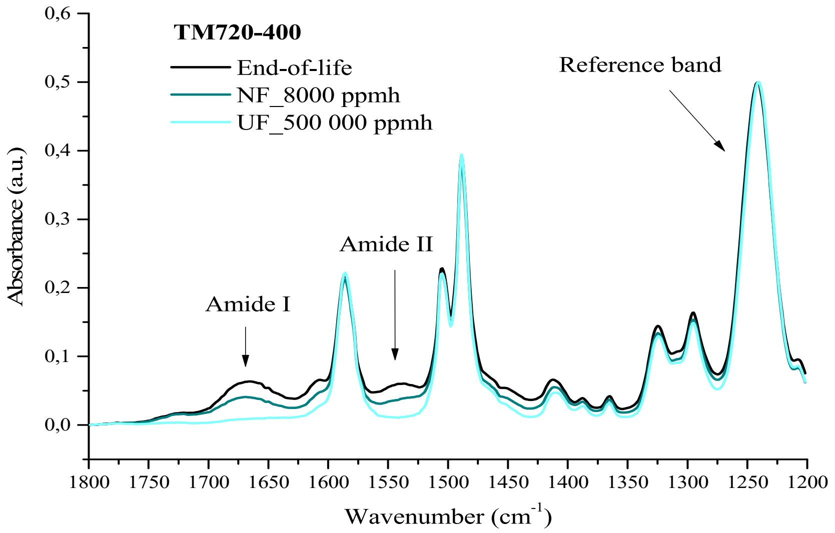

Figure S1. Attenuate total reflectance-Fourier transform infrared comparison images to verify the end-of-life RO membrane transformation.

It can be observed in Fig. S1 that the ATR-FTIR spectra of the membranes after being exposed to the NaOCl solution changed with respect to the pristine state (end-of-life membrane). These results indicate that these treatments were successfully achieved. For the transformation to nanofiltration membranes (dark green) the signals equivalent to the Amide I and Amide II groups can be detected with lower intensity compared with the end-of-life RO membranes. In contrast, for the transformation to ultrafiltration membranes (light green) no signals equivalent to the Amide I and Amide II groups can be detected, which means that the thin polyamide layer was completely removed.

S2. Scanning electron microscopy

Scanning Electron microscopy (SEM) using the S-8000 Model (Hitachi) image device was employed to observe the surface morphologies of the membranes and their stability in the implementation in ED to treat UWW. Preceding the microscopy analysis, the membrane samples were dried by heating at 50 ºC for 48 hours and later were gold-sputtered with a Sputter Coater Polaron SC7640 model to achieve 13–15 nm thickness prior to the SEM analysis. Surface SEM microphotographs of rAEM samples in the pristine state and after ED treatment are presented in Fig. S2. The nature of this recycled membranes manufacturing technique results in heterogeneous anion-exchange membranes, obtaining a dense and rough surface, with a considerably homogeneous distribution of ion-exchange resin particles.

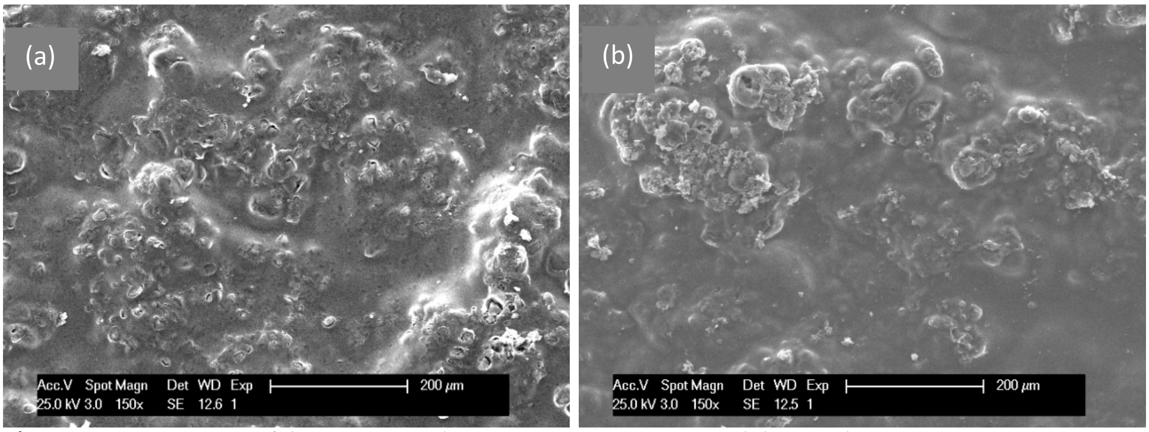

Figure S2. SEM images of the rAEM membranes (a) pristine rAEM, and (b) tested rAEM.

As can be observed in Fig. S2, the membrane after being used in UWW treatment did not present a salt deposition on its surface. Although, the long-term stability of the rAEMs in UWW treatment by ED should be addressed in future studies.

S3. Guideline of the water quality for irrigation

Table S2 summarizes the main parameters of the quality of reclaimed water for irrigation purposes regulated by the World Health Organization (WHO).

Table S2. WHO guidelines of wastewater reused for irrigation [2]

**Parameter**		**Unit**	**Degree of restriction on use**		
			**None**	**Slight to moderate**	**Severe**
**SAR**	=0 – 3	EC =	>700	700 – 200	<200
	=3 – 6	EC =	>1200	1200 – 300	<300
	=6 – 12	EC =	>1900	1900 – 500	<500
	=12 – 20	EC =	>2900	2900 – 1300	<1300
	=20 – 40	EC =	>5000	5000 – 2900	<2900
**Na^+**	Surface irrigation	mg L^{-1}	<69	69 – 207	>207
	Sprinkler irrigation	mg L^{-1}	<69	>69	
**Cl^-**	Surface irrigation	mg L^{-1}	<142	142 – 354	>354
	Sprinkler irrigation	mg L^{-1}	<106.5	>106.5	
**Total Nitrogen (TN)**		mg L^{-1}	<5	5 – 30	>30

**Nitrogen (NO_3-N)**	mg L^{-1}	<5	5 – 30	>30
**pH**	-		6.5 – 8 (8.5)	
**Anions and cations**				
**NO_2^-**	mg L^{-1} (meq L^{-1})		-	
**SO_4^{2-}**	mg L^{-1} (meq L^{-1})		960 (20)	
**Mg^{2+}**	mg L^{-1} (meq L^{-1})		61 (5)	
**Ca^{2+}**	mg L^{-1} (meq L^{-1})		400 (20)	
**Nutrients**				
**NO_3^-**	mg L^{-1} (meq L^{-1})		140 (10)	
**NH_4^+**	mg L^{-1} (meq L^{-1})		90 (5)	
**PO_4^{3-}**	mg L^{-1} (meq L^{-1})		194 (2)	
**K^+**	mg L^{-1} (meq L^{-1})		78 (2)	

S4. Lettuce crops

The evaluation of the quality of the reclaimed water by the irrigation lettuces was conducted in a growth cabinet of 1 m^3 size. The distribution of the pots, inside the cabinet, was arranged in a completely randomized design as it is shown in Fig. S3.

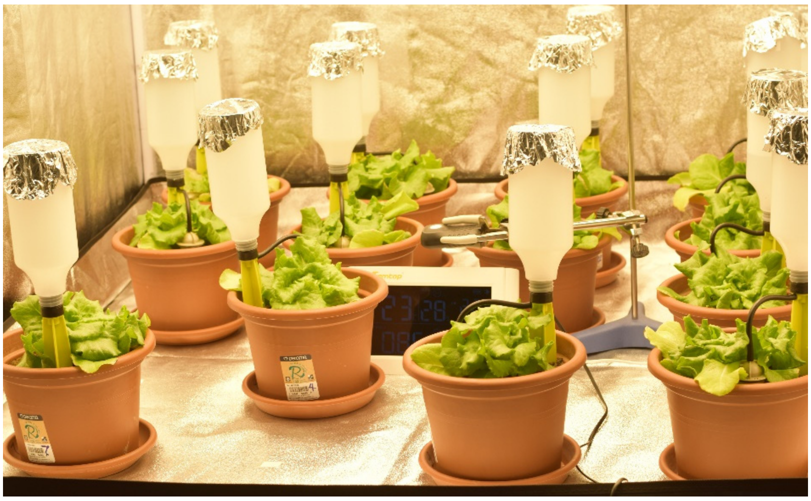

Figure S3. Picture of the lettuce crops in the fourth cultivation week.

An indoor design was selected for our study to compare the quality of the applied treatment, minimizing the influence of the variations of the environmental parameters (i.e., temperature, humidity, photoperiod, and photosynthetically active radiation).

Fig. S4 shows the harvested plants after a cultivation period of 49 days. The shoots were cut from each plant at the soil level.

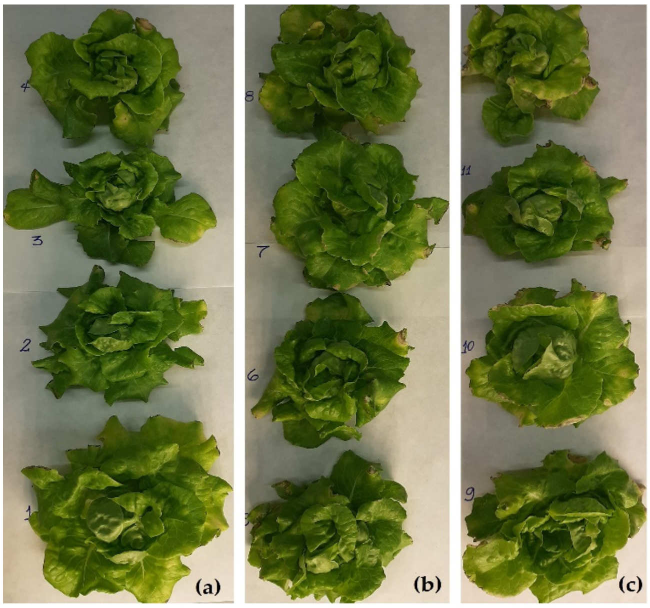

Figure S4. Harvested plants after 49 days. (a) irrigation with fresh water (TW), (b) irrigation with reclaimed water by rNF membrane (IRR), and (c) irrigation with reclaimed water by ED using rAEM (FRT).

Dendrometry parameters were measured in each plant, such as their head diameter and weight. It was not possible to count the number of leaves due to the rounded-head shape.

References:

[1] García-Pacheco, R. Nanofiltration and Ultrafiltration Membranes from End-of-Life Reverse Osmosis Membranes: A Study of Recycling, **2017**.

[2] Racar, M.; Dolar, D.; Karadakić, K.; Čavarović, N.; Glumac, N.; Ašperger, D.; Košutić, K. Challenges of Municipal Wastewater Reclamation for Irrigation by MBR and NF/RO: Physico-Chemical and Microbiological Parameters, and Emerging Contaminants. *Sci. Total Environ.* **2020**, *722*, 137959, doi:10.1016/J.SCITOTENV.2020.137959.
